# Identification of drug candidates against glioblastoma with machine learning and high-throughput screening of heterogeneous cellular models

**DOI:** 10.1039/d5dd00190k

**Published:** 2026-05-13

**Authors:** Vanessa Smer-Barreto, Richard J. R. Elliott, John C. Dawson, Álvaro Lorente-Macías, Muhammad Furqan, Asier Unciti-Broceta, Diego A. Oyarzún, Neil O. Carragher

**Affiliations:** a Cancer Research UK Scotland Centre, Institute of Genetics and Cancer, University of Edinburgh Crewe Road South Edinburgh EH4 2XR UK d.oyarzun@ed.ac.uk; b School of Informatics, University of Edinburgh 10 Crichton St Edinburgh EH8 9AB UK n.carragher@ed.ac.uk; c School of Biological Sciences, University of Edinburgh Max Born Crescent Edinburgh EH9 3BF UK

## Abstract

Glioblastoma multiforme (GBM) is an aggressive primary brain tumour that presents significant treatment challenges due to its complex pathology and heterogeneity. The lack of validated molecular targets is a major obstacle for discovering new therapeutic candidates, with no new effective GBM therapies delivered to patients in over two decades. Here, we report the identification of compounds that target the GBM stem cell survival phenotype. Our approach employs machine learning (ML) predictors of cell survival trained on high-throughput, image-based, phenotypic screening data for 3561 compounds, at multiple concentrations, across a panel of six heterogeneous, patient-derived, GBM stem cell lines. We computationally screened more than 12 000 compounds spanning various chemical classes. Experimental validation of ML-identified candidates across the GBM stem cell lines led to the identification of three compounds with activity against the GBM phenotype. Notably, one of our validated hits, the HSP90 inhibitor XL-888, displayed targeted elimination of all six GBM stem cell lines with IC_50_ in the nanomolar range. Further analyses suggest an XL-888 mechanism of action based on competitive ATP inhibition of HSP90 followed by disruption of HSP90 client proteins, and identify XL-888 as a promising candidate for future personalised medicine campaigns. Our work demonstrates that the use of phenotypic screening in tandem with ML can effectively identify therapeutic leads for personalised treatments in highly heterogeneous indications with few known molecular targets.

## Abstract 2

Glioblastoma multiforme (GBM) is an aggressive primary brain tumour that presents significant treatment challenges due to its complex pathology and heterogeneity.

## Graphical abstract

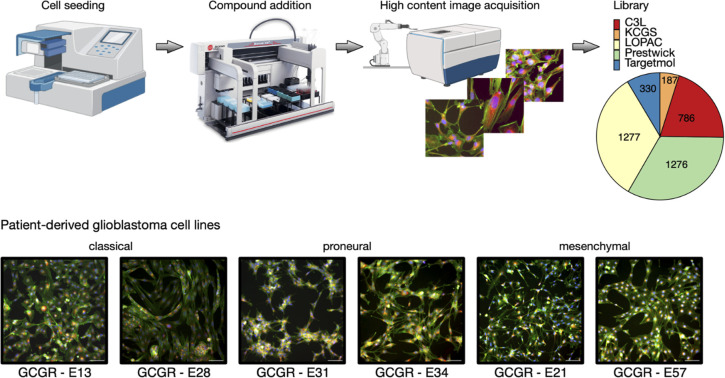

## Introduction

Glioblastoma multiforme (GBM) is the most common and aggressive primary brain tumour found in human adults and is characterised by substantial heterogeneity of genetic drivers and the tumour microenvironment.^1–3^ Patients have poor prognosis and limited treatment options (typically surgery followed by chemoradiation) that lead to the emergence of resistance. For the past 20 years, the standard of care for newly diagnosed GBM patients has consisted of surgery, temozolomide (TMZ), and ionizing radiation (IR) prolonging the median overall survival of patients from 12 to 15 months.^4,5^ Large-scale genomic analyses have enhanced our understanding of the molecular biology of GBM, which has supported the classification of GBM into various subtypes.^6–8^ However, this new understanding of GBM has not yet delivered new effective treatment strategies. Current trials are limited to narrow subtypes of the disease characterised by well established druggable target pathways that have failed to demonstrate durable responses.^9,10^

The dismal prognosis of this cancer of unmet need urgently calls for innovative approaches to find early drug candidates that can overcome the challenges of heterogeneity, including GBM stem cell plasticity and drug resistance, as well as effectively permeate the blood–brain barrier. The intratumoural heterogeneity and ability of GBM stem cells to rapidly rewire signalling networks in response to inhibition of a single pathway^11^ confounds modern target-based drug discovery strategies. As a result, several studies have explored the use of target-agnostic phenotypic screening in GBM models, with particular efforts on the application of patient-derived cell lines,^12–14^ drug repurposing,^1,15^ and droplet microarray platforms.^16^

Computational screens based on Artificial Intelligence (AI) have recently gained notoriety due to their ability to sieve through vast collections of chemical data at scale, detecting patterns to identify compounds with a high probability of displaying a therapeutic phenotype of interest.^17,18^ Machine learning models trained on phenotypic data have delivered a number of recent successes, including the discovery of senolytics,^19^ novel antibiotics,^20^ immune modulators,^21^ and anti-inflammatory leads,^22^ and constitutes one of the most exciting avenues for early drug discovery applications. The effectiveness of phenotypic- and AI- driven drug discovery depends on the availability of high-quality data acquired on disease-relevant model systems. In the case of GBM, it is possible to culture and propagate patient-derived GBM stem cells under conditions that maintain their stem cell like properties, thus recapitulating the heterogeneity and drug resistance mechanisms that lead to relapse in patients.^23^

In this work, we employed high-throughput phenotypic screening data and ML for discovering small molecule leads that hold potential for the development of alternatives to TMZ treatment of GBM. Using an in-house high-throughput screening assay of six patient-derived GBM stem cell lines,^24^ we trained ML algorithms and successfully validated three predicted hits in cell lines representing the three main transcriptomic subtypes of GBM (proneural, classical, and mesenchymal). Further analyses identified our hit XL-888 as a promising starting point for chemical optimization campaigns. Our data suggest that XL-888 targets GBM stem cell survival and proliferation *via* competitive ATP inhibition of HSP90 followed by disruption of HSP90 client proteins implicated in oncogenic signalling resulting in cell cycle arrest and apoptosis.

Artificial intelligence and ML have been successfully applied in the context of GBM for drug repurposing,^1^ tumour identification,^25–27^ patient survival prediction,^28–30^ and biomarker prediction.^31,32^ To the best of our knowledge, our study is the first reporting the AI-powered discovery of chemical leads against the highly heterogeneous GBM phenotype. This demonstrates the power of data-driven methods in indications with poorly understood target biology, and highlights the benefits of employing training data that captures the heterogeneity of the disease of interest.

## Results

### High-throughput glioblastoma screen

**Fig. 1:**
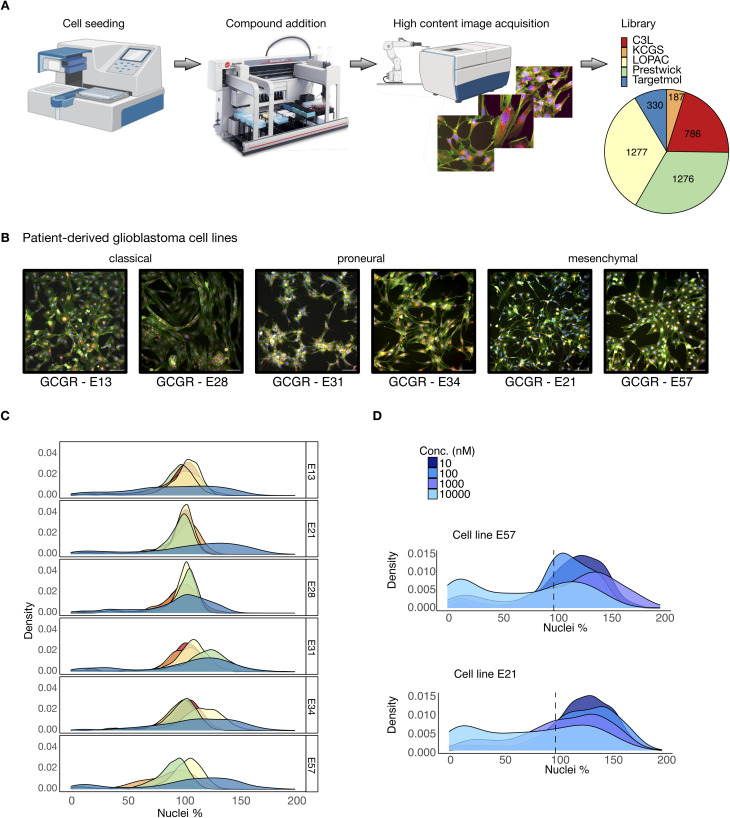
High-throughput phenotypic assay screening of heterogeneous, patient-derived GBM cell lines. (A) High content phenotypic assay screening workflow on GBM cells.^24^ Five libraries were screened at multiple concentrations (C3L, 786 compounds at [0.003, 0.03, 0.3, 3] µm; KCGS, 187 compounds at [0.1, 1] µm; LOPAC, 1277 compounds at [0.5, 3] µm; Prestwick, 1276 compounds at [1, 10] µm; TargetMol-330, 330 compounds at [0.01, 0.1, 1, 10] µm). (B) Representative images of multiplex cell painting assay screen performed across the three main GBM cellular subtypes: classical (GCGR-E13, GCGR-E28), proneural (GCGR-E31, GCGR-E34), and mesenchymal (GCGR-E21, GCGR-E57). The cell painting stains shown are Hoechst (blue), phalloidin/WGA (green), and endoplasmic reticulum (red). (C) Density plots over all six GBM cell lines of the percentage of nuclei counts after compound treatment with respect to the average DMSO negative control (100%) per plate. After removal of duplicates, the dataset contains 3561 unique compounds. Colours correspond to each library (panel A), and only the highest concentration per library is shown. Cell line sensitivity to the compounds is variable across the cell lines; the bimodality displayed by the TargetMol library reveals a larger fraction of compounds that affect cell viability as compared to the other four libraries. (D) Density plots of the TargetMol library screen at all concentrations of cell line GCGR-E57 (top) and of cell line GCGR-E21 (bottom). The black dotted line represents the average DMSO negative control nuclei count. As the concentration increases, a larger number of compounds affects cell viability.

The screening protocol across a panel of patient derived GBM stem cells is represented in Fig. 1A, with further detail provided in the Methods section and in Elliott *et al.*^24^ We employed the ImageXpress-confocal Ht.ai high content imaging platform (molecular devices) to quantify cell nuclei counts following small-molecule compound treatments as a readout for cell viability across six fields-of-view for each compound treatment (20× objective). We performed our assay using six genetically diverse patient-derived glioma stem cell lines GCGR-E13, GCGR-E21, GCGR-E28, GCGR-E31, GCGR-E34, and GCGR-E57, two from each of the most prominent subtypes of glioblastoma: classical, proneural, and mesenchymal (Fig. 1B). Our screen included a total of 3561 compounds at variable concentrations (Fig. 1A) drawn from the Comprehensive anti-Cancer small Compound Library^33^ (C3L), the Kinase Chemogenomic Set (KCGS), the Library of Pharmacologically Active Compounds (LOPAC), the Prestwick chemical library, and the TargetMol anticancer library. We employed DMSO (0.1% v/v) as negative control and staurosporine (STS) and/or paclitaxel (PAC) as positive controls at concentrations of 1 µM STS (C3L, LOPAC, Prestwick chemical library), 0.1 µM PAC (KCGS) and both 5 nM PAC and 0.1 µM STS (TargetMol anticancer library). These libraries were chosen to represent selectivity and potency against known oncology and kinase targets, and the inclusion of the broad pharmacological diversity of approved small molecule compounds. The library is therefore biased towards existing drug targets and pharmacological classes, and is therefore limited in representation of broader search spaces.

We observed variations in the proportion of compounds that affected target cell viability, both across cell lines and compound libraries, and this variation appeared to increase at higher concentrations (Fig. 1C). The density of compounds' cell survival against DMSO control at the maximum concentration per library (C3L – 3 µM, KCGS – 1 µM, LOPAC – 3 µM, Prestwick – 10 µM, and TargetMol – 10 µM) shows a bimodal distribution for the TargetMol library, with more treatments that affect the cells' viability. In contrast, the LOPAC library's unimodal distribution is centred on the DMSO control value of 100%. We further observed a higher susceptibility in some cell lines, as is the case for GCGR-E57, which displays a larger bimodal effect as the concentration increases on the TargetMol library screen (Fig. 1D top panel) in comparison to GCGR-E21 for the same conditions (Fig. 1D bottom panel).

### Machine learning models of GBM cell viability

We employed our screening data to train machine learning models of GBM cell viability in response to compound treatment, using a binary classification approach. Since our screen was performed at multiple concentrations and these varied across libraries, we labelled compounds as positive (leading to GBM cell death) or negative using a bespoke concentration-sensitive approach. This allowed us to make maximal use of the compound data and integrate all compounds into a single labelled dataset for model training. To this end, for each compound we first chose the concentration for which the phenotypic response was closest to 50% of nuclei count.

Compounds were then labelled as positive if they eliminated at least two and not all six GBM cell lines using a cutoff of 35% nuclei count survival with respect to negative, DMSO controls. Our threshold of 35% for compound labelling was informed by the performance of the positive control FDA-approved drug paclitaxel at 5 nM, whose mean nuclei count is equivalent to a 36% nuclear count survival rate relative to DMSO negative control. This cutoff was chosen because we aim to find compounds that cause positive control-like levels of cell viability damage in GBM cells and mitigate against bias toward general cytotoxic compounds with a narrow therapeutic index. Details on our compound labelling pipeline can be found in the Methods.

**Fig. 2:**
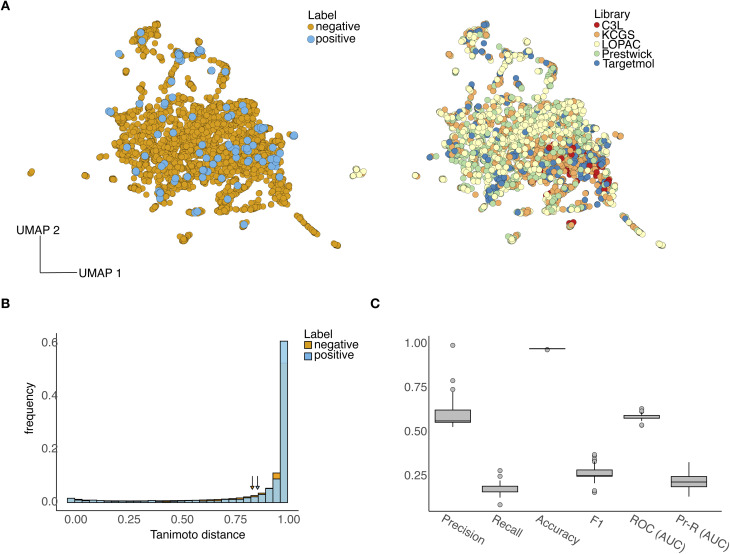
Visualisation of chemical space and training of machine learning models. (A) Two-dimensional UMAP visualisation of the 3561 compounds employed for training machine learning models. Compounds were represented with a feature vector of 200 physicochemical descriptors computed with the RDKit^34^ software. UMAP plots were generated with number of neighbors 50, minimum distance 0.1, and spread 1. To train binary classifiers, the dataset was labelled as positives (compounds that eliminated 35% or more of the cells in the wells, and that did so for more than two but not all sixe cell lines) and negatives (all other compounds). Compounds were coloured according to their binary labels (left) and library of origin (right). (B) Tanimoto distances between compounds in the training set, split according to positives (*N* = 10 506, all pairs) and negatives (*N* = 11 954 298 pairs). The average Tanimoto distance across the positives is 0.88 ± 0.25; for the negatives cohort is 0.85 ± 0.26, suggesting suitable chemical diversity in both compound classes. Tanimoto distances were computed on RDKit features after normalization to zero mean and unit variance. (C) Performance metrics of 100 XGBoost algorithms trained on Monte Carlo re-sampled sets of 70% of compounds and tested on the remaining 30% (see Methods). Performance metrics include precision, recall, accuracy, *F*1 score, and area under the curve (AUC) of both receiver operating characteristic and Precision–Recall curves. Box-and-whisker plots show the interquartile range and outliers across the 100 models, all of which surpass the naive accuracy baseline given by the class imbalance (2.9%).

For model training, we featurized each compound with physicochemical descriptors calculated with RDKit,^34^ which have been successfully employed in previous models trained on phenotypic data.^19,35^ The resulting dataset contains 103 positive and 3458 negative compounds for training, and is well distributed in the physicochemical feature space (Fig. 2A); low-dimensional representations using the UMAP algorithm^36^ do not suggest a per-library bias (Fig. 2A, left) and a suitable degree of chemical diversity for model training (Fig. 2A right). The distribution of pairwise Tanimoto distances (Fig. 2B) within positive and negative compounds suggests appropriate chemical diversity and reinforces the qualitative observations from the UMAP representations (Fig. 2A).

Given the strong class imbalance in the data (2.9%), we devised a strategy to maximise the use of positive samples and, at the same time, yield confident predictions for downstream hit validation. To this end, we employed a Monte Carlo approach whereby a binary classifier was trained and tested multiple times with stratified resampled training (70%) and test data (30%), from where a panel of 100 models with performance above a baseline were selected for virtual screening. This strategy ensures that all positives are employed for training in the Monte Carlo loop, and produces robust screening predictions through ensemble scoring of unlabelled compounds. As a base model we chose an XGBoost classifier, which produces predictions using an ensemble of decision trees. After performing feature selection and Monte Carlo retraining on resampled training sets containing 70% of labelled compounds (see Methods), we selected *N* = 100 models with a test accuracy above the naive baseline, whereby all test compounds are called as the majority class. The performance metrics of the selected models (Fig. 2C) show strong tradeoffs between precision (fraction of true positives out of all positive predictions) and recall (fraction of correct positive predictions), generally indicating modest model performance (Fig. 2C) as a result of the strong class imbalance (2.9%) and low number of positive samples for training (*N* = 103 positives).^19,37^ However, the XGBoost models scored an encouraging precision of 0.6 ± 0.08 across the *N* = 100 Monte Carlo runs, which we deemed suitable for prioritizing compounds for experimental hit validation and reducing false positives.

### Computational screen and hit validation against the GBM phenotype

**Fig. 3:**
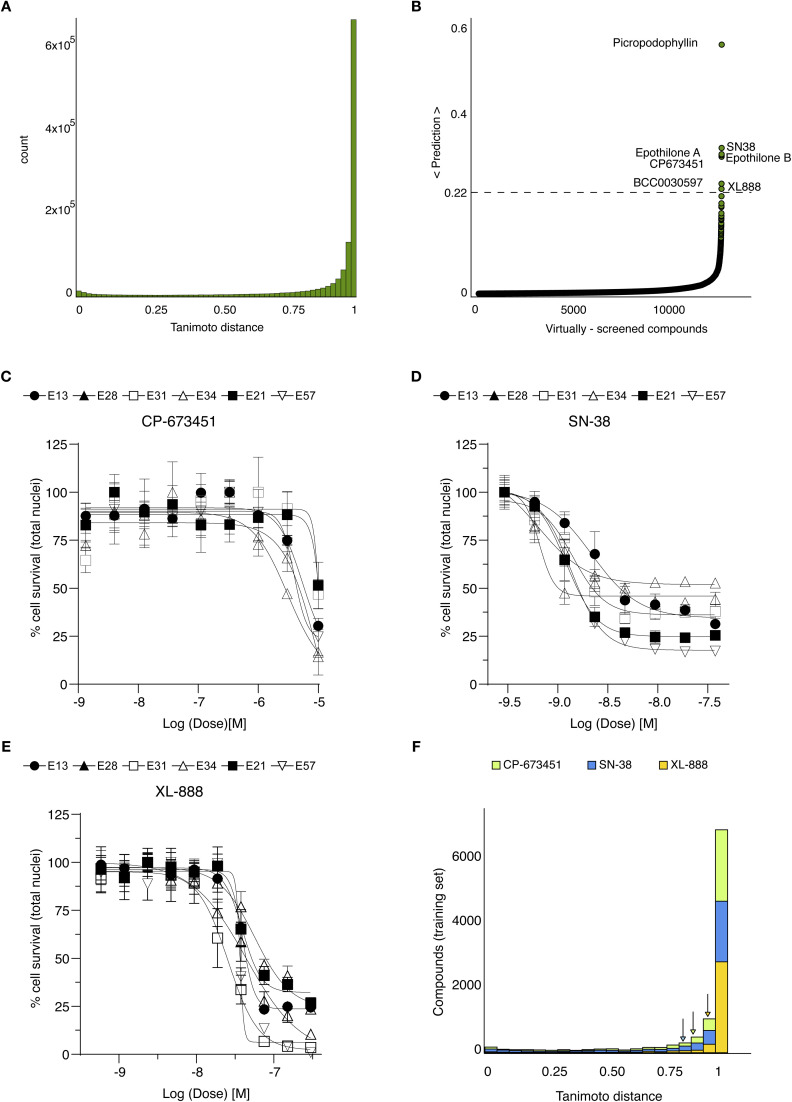
Computational screening and hit validation in GBM cellular models. (A) Compounds from two libraries, TargetMol-3000 and BioAscent, were selected for computational screening, giving a total of 12 888 unique compounds. The histogram shows the Tanimoto distance from each computationally-screened compound to each of the 103 positives in the training set; average Tanimoto distance is 0.86 ± 0.25 suggesting good diversity with respect to positive in training. (B) Computational screen of 12 888 compounds from the TargetMol-3000 and BioAscent libraries. Histogram shows the predicted probability of each compound affecting viability of GBM cellular models. The predictions were calculated for each of the 100 XGBoost models; histogram shows average prediction score for each compound across the trained models. Model predictions were highly selective, with only seven compounds scoring above 22%. Three compounds were not selected for hit validation because of closely-related analogues in the training set. Four compounds were taken forward for experimental validation in all six GBM cells lines. (C–E) Dose–response curves of three validated hits that affect the cell viability of GBM cell lines. Plots show dose–response curves and chemical structures of (C) CP-673451, (D) SN-38, and (E) XL-888. SN-38 and XL-888 are effective at the nanomolar concentration range, while CP-673451 is active at micromolar concentrations. Mean ± s.d. are shown from *n* = 3 experiments. (F) Stacked histograms of the Tanimoto distance between the three validated hits and each of the 3561 compounds in the training set; average Tanimoto distance was 0.88 ± 0.24 for CP-673451, 0.83 ± 0.28 for SN-38, and 0.94 ± 0.17 for XL-888, indicating substantial physicochemical differences between the hits and the compounds for training.

We employed the ensemble of machine learning models for virtual screening and experimental testing of the predicted hits. For screening, we aggregated the anticancer TargetMol-3000 and Bioascent libraries. These libraries were chosen because the TargetMol library represents a collection of structurally diverse, medicinally active, and cell permeable FDA approved and clinical stage drug candidates each associated with rich documentation on structure, target, IC_50_ value and biological activity description. The Bioascent library contains broader structural diversity of an accessible 120 000 parent library of lead-like small molecules which are commercially available for follow up studies. After removal of duplicates and compounds present in the training data, we obtained a total 12 888 unique compounds for our screening library, with good diversity with respect to the positives in the training data (Fig. 3A).

After querying the ensemble of machine learning models, we obtained highly selective prediction scores (Fig. 3B), with only seven predicted hits (0.05%) with an average score above 22%. This high selectivity is two orders of magnitude below the class imbalance of the training data and adds confidence in model predictions. Examination of the predicted hits revealed that three compounds (epothilone A, epothilone B, and picropodophyllin) have close analogues in the training data (ixabepilone and podophyllotoxin), and thus were excluded from experimental testing. We tested the remaining four predicted hits in our GBM cell lines with a similar assay as for our high-content screening (Fig. 1A). Among the tested hits, three compounds (CP-673451, SN-38, and XL-888) affected the viability of GBM cell lines (Fig. 3C–E, Table 1 and SI Fig. 1): CP-673451 is a potent and selective inhibitor of platelet derived growth factor (PDGFR); SN-38 is the active metabolite of irinotecan, an approved topoisomerase inhibitor; XL-888 is a known inhibitor of Heat shock protein 90 (HSP90). The remaining tested compound (BCC0030597) did not impact GBM cell viability. The validated compounds displayed varied activity against the GBM phenotype (Table 1) with SN-38 and XL-888 having IC_50_ below 10 µM for all six cell lines, while CP-673451 only for a subset of them, possibly due to genetic heterogeneity conferring distinct sensitivity between cell lines (Fig. 3C–E).

**Table 1:** IC_50_ Profiles of XL-888, SN-38, CP-673451 across 6 patient derived glioma stem cell lines. Cell lines are grouped by GBM sub-type, IC_50_ values are in nanomolar (nM) with associated 95% confidence interval (CI) range (*n* = 3). Compound structures are shown together with their targets

Sub-type	Cell line	Compound	IC_50_ nM [95% CI range]		
			XL-888	SN-38	CP-673451
Classical	E28	IC_50_	44	4.6	3520
		95% CI	39–49	2.9–7.2	2037–6968
	E13	IC_50_	56	6.6	5850
		95% CI	49–65	5.4–8.2	4384–8053
Mesenchymal	E21	IC_50_	82	2.4	>10 000
		95% CI	71–95	1.9–3.0	Wide
	E57	IC_50_	35	2.1	5237
		95% CI	32–39	1.6–2.7	3906–7106
Proneural	E34	IC_50_	94	9.8	2552
		95% CI	83–96	6.2–15	1707–3884
	E31	IC_50_	25	4.1	>10 000
		95% CI	22–27	3.0–5.6	Wide
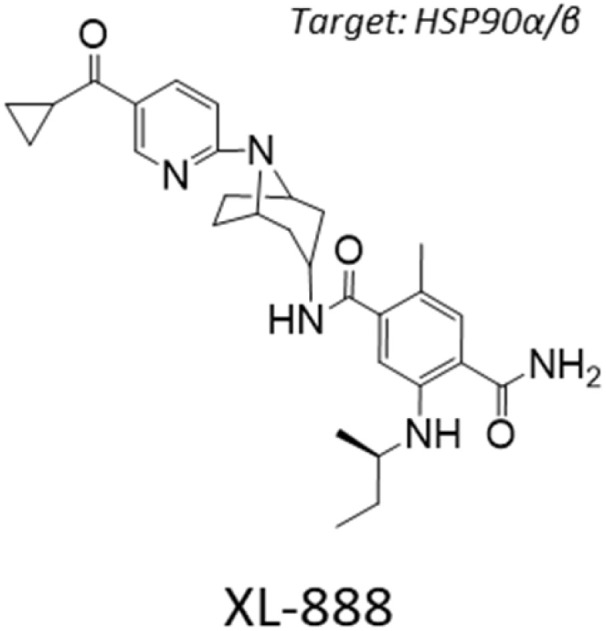		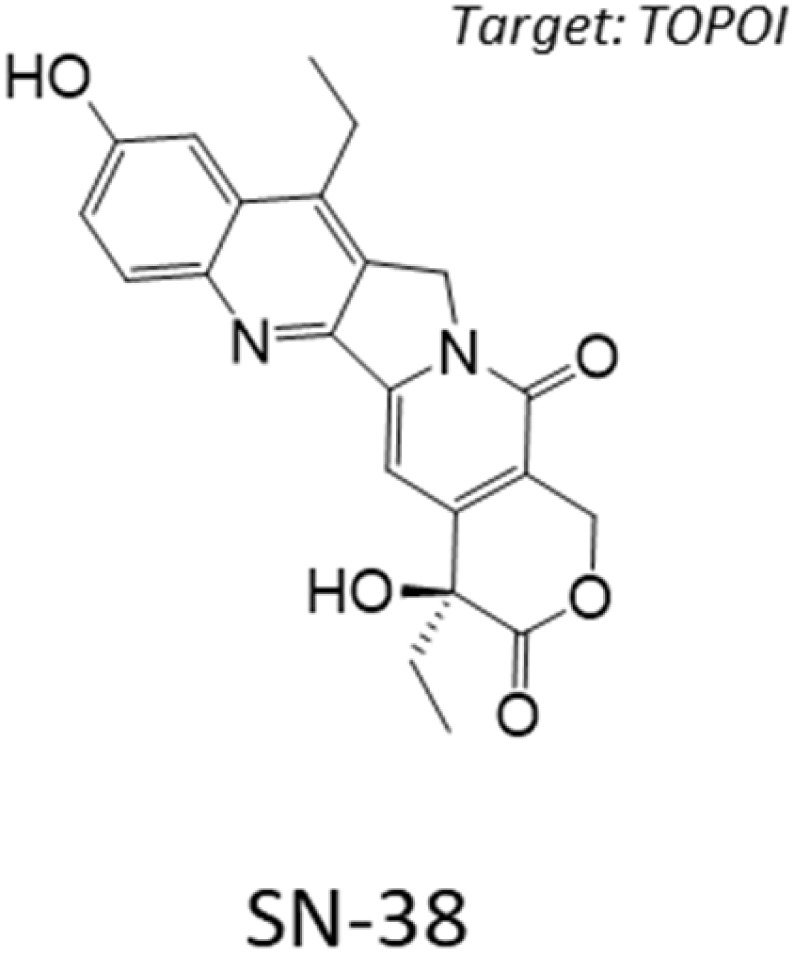		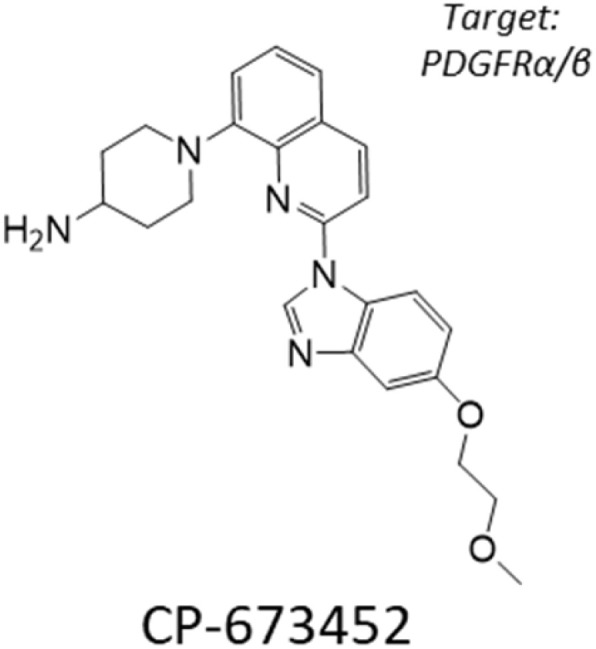	

**Fig. 4:**
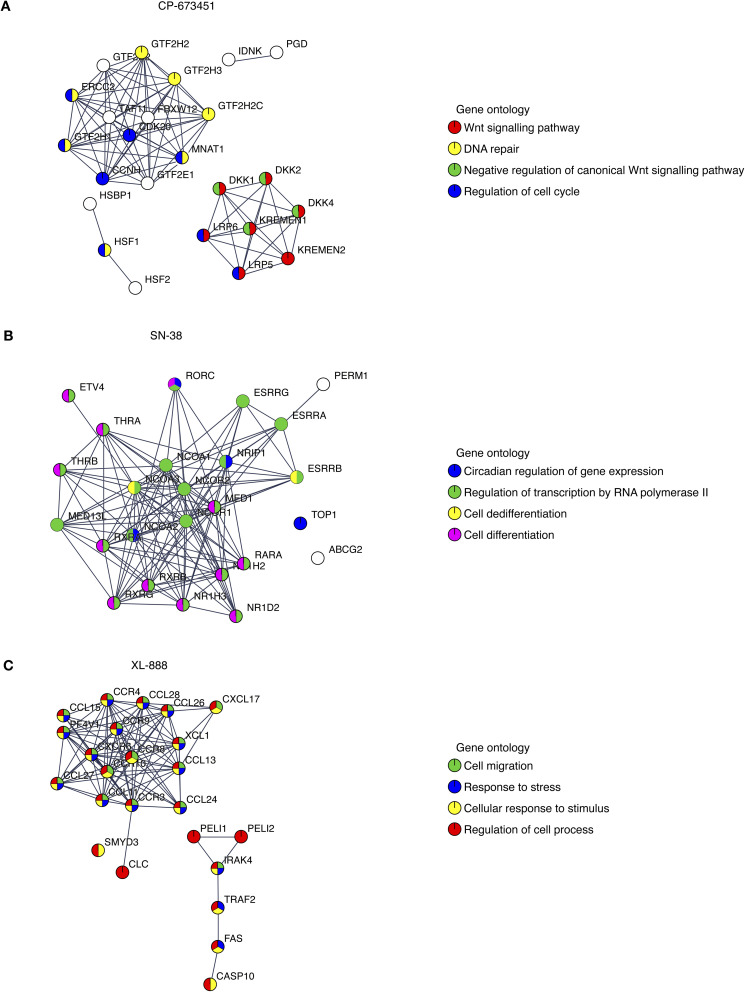
STRING pathway network analysis. (A–C) STRING analysis of putative intracellular pathway signalling networks influenced by each of the three compounds identified by the computational screen and characterised experimentally: (A) CP-673451, (B) SN-38, and (C) XL-888. Gene ontology terms are highlighted for each network.

We further investigated the potential mechanism-of-action of the validated hits *via* STRING-Cytoscape analysis^38^ and similarity ensemble approach (SEA^39^), which showed enrichment for cell cycle regulation, gene expression, and chemokine signalling targets (Fig. 4A–C).

### Mechanistic investigation of GBM candidate XL-888

**Fig. 5:**
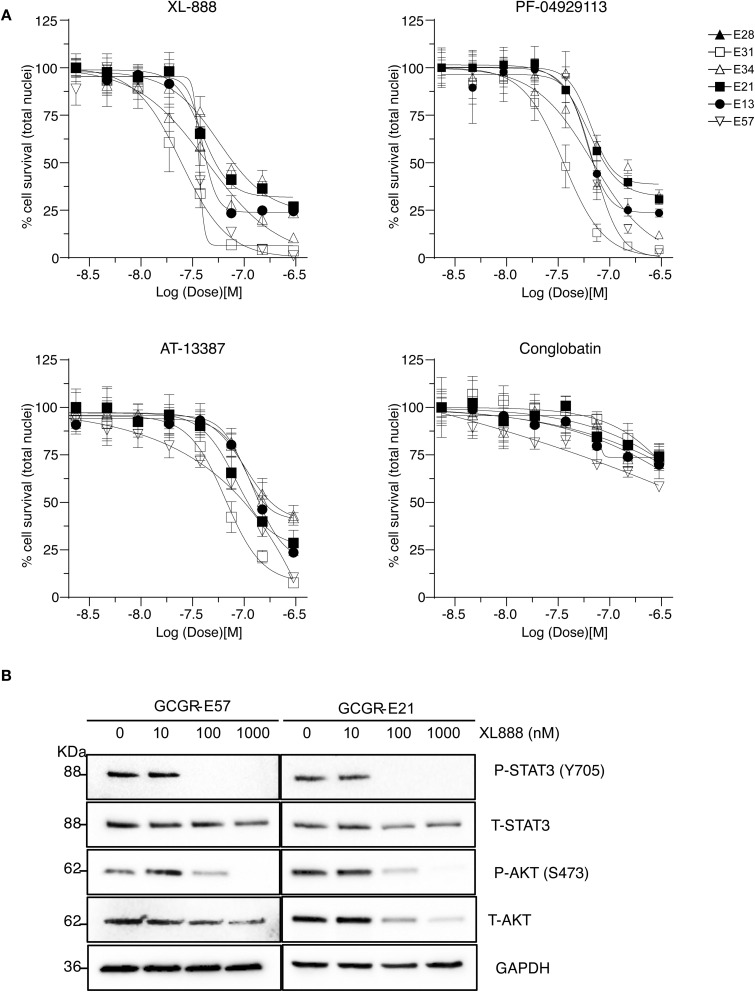
Mechanism of action for XL-888. (A) Dose response profiles of structurally and mechanistically distinct HSP90 inhibitors across the GBM stem cell panel. (B) Western blot analysis of total and phosphorylated proteins in key signalling pathways downstream of HSP90.

Out of our three candidates, XL-888 is the most promising because it has reached clinical trials for treating solid malignancies in combination with targeted therapies.^40,41^ XL-888 has previously been reported to bind to the ATP-binding site of HSP90 ^42^ and thus belongs to the class of N-terminal ATPases HSP90 inhibitors that competitively bind to ATP binding sites to disrupt the ability of HSP90 to hydrolyze ATP and perform its chaperone functions. The chemical structure of XL-888 is remarkably different to that of other HSP90 inhibitors, further increasing its value as a novel candidate drug for GBM treatment (Table 2). To elucidate the mechanism-of-action of XL-888 mediated inhibition of glioblastoma stem cell survival, we tested XL-888 and two structurally distinct N-terminal ATPase HSP90 inhibitors (AT-13887 and PF-0429113) as well as an alternative HSP90-Cdc37 complex inhibitor (conglobatin) across our panel of 6 GBM stem cell lines (Fig. 5A and Table 2). XL-888, AT-13887, and PF-0429113 displayed similar potency profiles across our panel of 6 GBM stem cell lines. In contrast, conglobatin which binds to a specific domain of HSP90 to disrupt HSP90-Cdc37 complex formation, which is required for activation and maturation of HSP90 client proteins such as K-Ras and HIF1α, demonstrates only weak activity across the GBM stem cell panel. We then performed western blot analysis of two HSP90 client proteins and downstream signalling pathways, Akt and STAT3, which are known to drive GBM cell proliferation and survival in cell lines GCGR-E21 and -E57 (Fig. 5B). We found that XL-888 results in a reduction in both the abundance and phosphorylation of these two key HSP90 client proteins.

**Table 2:** IC_50_ profiles of structurally distinct HSP90 inhibitors across 6 patient derived glioma stem cell lines. Cell lines are grouped by GBM sub-type, IC_50_ values are in nanomolar (nM) with associated 95% confidence interval (CI) range (*n* = 3). Compound structures are shown with annotated cellular targets [note: data for XL-888 reproduced here for direct comparison]

Sub-type	Cell line	Compound	IC_50_ nM [95% CI range]			
			AT-13387	Conglobation	PF-04929113	XL-888
Classical	E28	IC_50_	128	1200	68	44
		95% CI	113–146	646–3576	61–77	39–49
	E13	IC_50_	148	918	84	56
		95% CI	134–163	463–3759	72–97	49–65
Mesenchymal	E21	IC_50_	208	1019	121	82
		95% CI	181–244	578–2900	106–138	71–95
	E57	IC_50_	95	548	69	35
		95% CI	83–108	421–756	63–76	32–39
Proneural	E34	IC_50_	213	1359	145	94
		95% CI	184–254	802–3018	125–172	83–96
	E31	IC_50_	75	631	36	25
		95% CI	69–83	459–1073	32–39	22–27
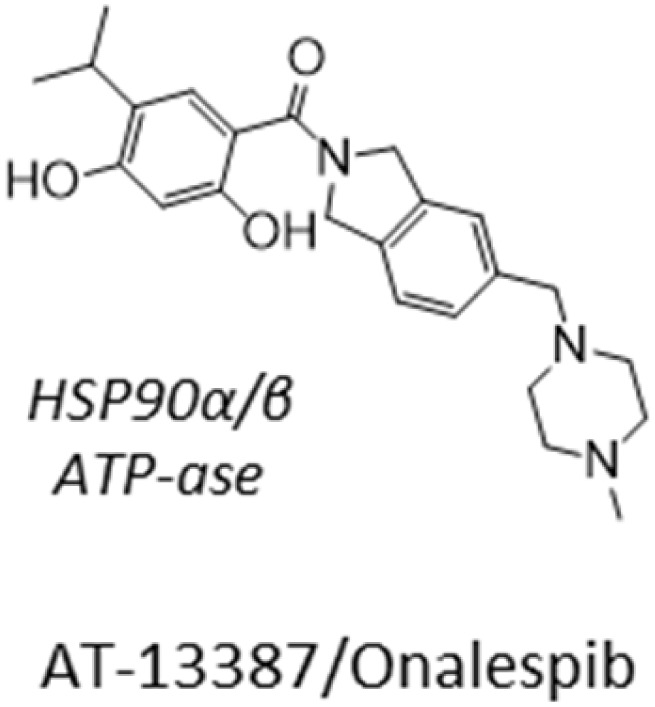	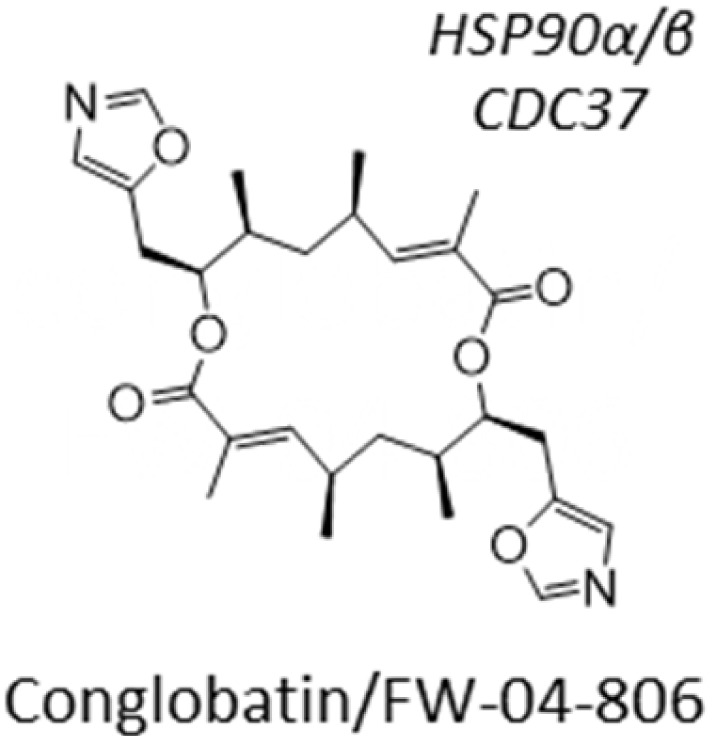		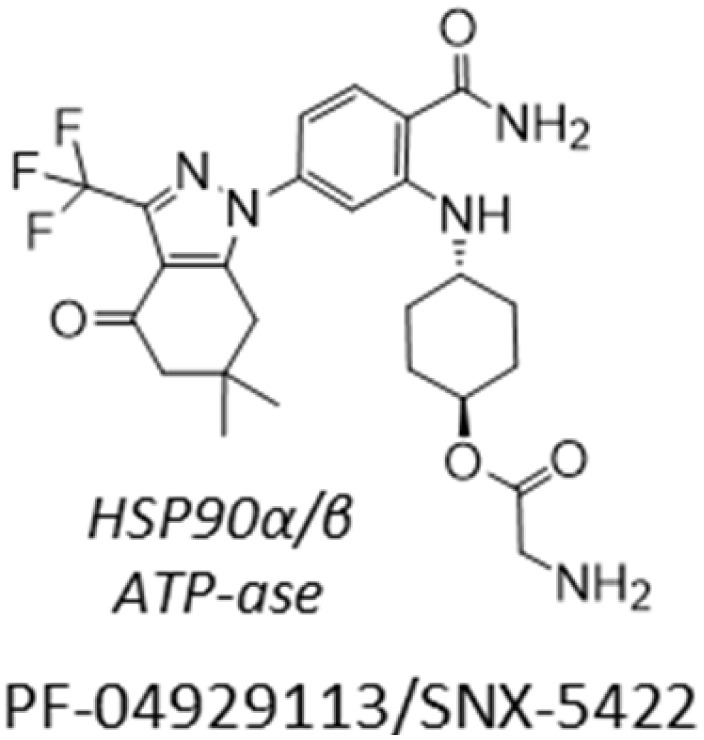		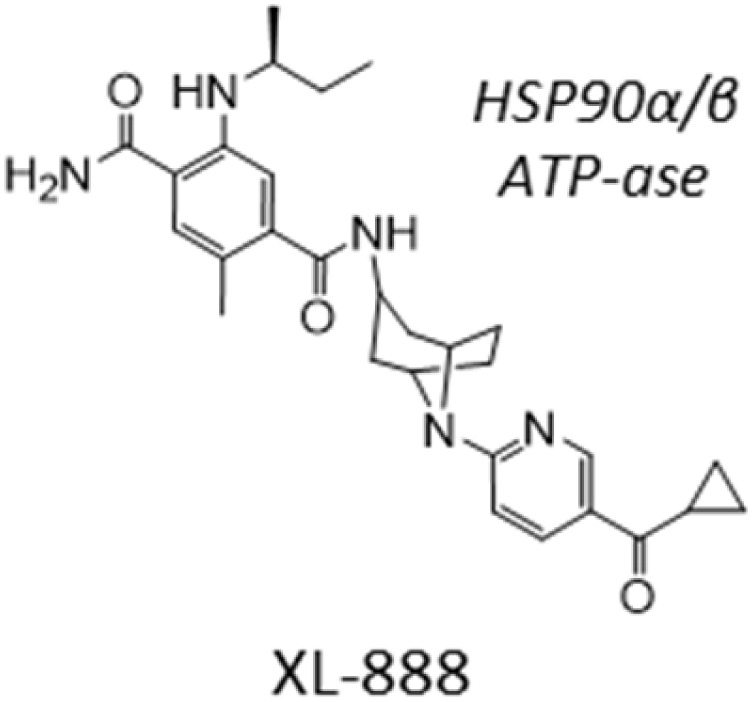	

After confirming the equivalence of profiles within structurally dissimilar N-terminal ATPase HSP90 inhibitors, we performed apoptosis and cell cycle analyses (SI Fig. 2 and 3) using a caspase activity biosensor and DNA content analysis respectively across XL-888 and AT-13887 in GBM stem cell lines GCGR-E21 and -E57. Consistent with their potency profiles and downstream pathway analyses, we found that both XL-888 and AT-13877 potently induce caspase mediated apoptosis (SI Fig. 2A and B) and a G2/M phase cell cycle arrest in the two GBM stem cell lines tested (SI Fig. 3A, B, 3D and E). The dose response of cell survival across the entire panel of 6 GBM stem cell lines demonstrates an increase in G2/M survival in most cases (SI Fig. 3C and F).

We further examined the impact of XL-888 treatment on the SRY-box transcription factor 2 (SOX2), an established stem cell marker and core component driving GBM stem cell renewal properties (SI Fig. 4A and B).^43,44^ We observed a dose-dependent reduction in SOX2 levels across the two GBM stem cell lines tested, GCGR-E21 and GCGR-E57, within 24 hours following treatment with XL-888. From a potency and medicinal chemistry perspective, we propose XL-888 as a promising chemical lead for further medicinal chemistry optimization towards the discovery of analogues with improved blood–brain barrier penetration. We suggest a general strategy (SI Fig. 5A and Table 1) for a GBM medicinal chemistry campaign on this compound. While XL-888 (MW = 503.64 Da) narrowly exceeds the molecular weight limit suggested by Lipinski's rules, in the drug discovery process many high throughput screening sets and lead-like molecules fall within the 400–650 Da range. This range provides sufficient chemical complexity and diversity to facilitate effective hit or lead identification.

## Discussion

Given the high attrition rates encountered in conventional drug discovery campaigns, particularly in complex disease areas of unmet need with poorly understood targets, there is growing interest in complementary avenues that can tackle this challenge at accelerated speed. Unbiased phenotypic screening in disease relevant models that are designed to recapitulate key segments of disease pathophysiology have been proposed as a strategy to reduce late-stage attrition in drug development.^45^ Drug screens performed in glioblastoma (GBM) models have so far been restricted to relatively small sets of target-annotated and approved drug libraries, limiting the potential for novel therapeutic discovery.^1^ Expanding screening to larger diverse chemical libraries in complex patient-derived models quickly becomes economically infeasible to most research laboratories. Artificial Intelligence and machine learning models provide a promising route to search large areas of chemical space with relatively small amounts of data.^19^

Here, we identified and validated three compounds against the GBM phenotype, using a carefully designed prediction pipeline trained on in-house data from patient-derived GBM stem cell lines. This diverse screen captures the intrinsic heterogeneity of the GBM phenotype that led to a robust dataset for model training and a substantially higher hit identification rate than standard experimental high-throughput screening. SN-38 is an active biological metabolite of the pro-drug irinotecan with primary mechanism-of-action annotated as a topoisomerase inhibitor. Topoisomerase inhibitors exhibit broad activity upon cancer cell survival^46^ and previous clinical studies have indicated that irinotecan when used either in combination with other compounds or following local delivery might improve outcome in patients with recurrent malignant glioma.^47,48^ An open-label Phase 2 safety and efficacy trial of irinotecan administered directly into the resection margin in patients with surgically resectable glioblastoma is currently underway.^49^ Our pathway network analysis indicates SN-38 may also be influencing the activity of several nuclear receptor coactivators and subfamily members such as Nuclear Receptor Subfamily 1 Group D Member 1 (NR1D1), which have previously been implicated as therapeutic targets in GBM.^50^ CP-673451 is a well characterized PDGFR inhibitor that has previously been demonstrated to promote GBM stem cell differentiation and inhibit GBM tumour growth *in vitro* and *in vivo*.^51^ Our pathway network analysis indicates that CP-673451 may also be potentially influencing GBM stem cell survival *via* modulation of heat shock factor 1 (HSF1), Kremen protein 1 (Kremen1) and General Transcription Factor IIH Subunit (GTF2H) subunit pathways. XL-888 is a potent inhibitor of HSP90 and thus exhibits a potential shared mechanism with CP-673451 inhibition of HSF1 which forms a complex with HSP90.^52^ HSP90 has previously been implicated in promoting GBM stem cell properties^53^ and HSP90 inhibitors such as 17-AAG demonstrate inhibition of GBM growth *in vitro* and *in vivo*.^54^ Our pathway analysis indicates XL-888 may also be modulating the GBM survival phenotype *via* perturbation of the chemokine^55^ and tumor necrosis factor receptor-associated factor 2 (TRAF2) signalling pathways previously implicated in GBM growth and radioresistance.^56^ To further elucidate the mechanism-of-action of XL-888 mediated inhibition of glioblastoma stem cell survival we tested a panel of structurally distinct N-terminal ATPase HSP90 inhibitors which revealed very similar potency profiles across our panel of 6 GSC lines. In contrast, conglobatin which binds to a separate domain on HSP90 to specifically disrupt complex formation between HSP90 and the co-chaperone cdc37 demonstrates only weak activity across the glioma stem cell panel. Furthermore reduction in both the abundance and phosphorylation of two HSP90 client proteins (Akt and STAT3) is observed following XL-888 treatment which is accompanied by induction of caspase mediated apoptosis and a G2/M phase cell cycle arrest across multiple GBM stem cell lines. Taken together these results are consistent with competitive ATP inhibition of HSP90 followed by disruption of HSP90 client proteins implicated in oncogenic signalling resulting in cell cycle arrest and apoptosis as the principal mechanism by which XL-888 is targeting GBM stem cell survival and proliferation.

SRY-box transcription factor 2 (SOX2) is a transcription factor that serves as a marker for neural stem cells, pluripotent cells in early development, and stem-like cancer stem cells from several tumour types including GBM.^43,57^ In GBM stem cells, SOX2 has been shown to promote self-renewal *via* transcriptional control of core cell cycle and epigenetic regulators.^44^ SOX2 has previously been shown to be a client protein stabilized by HSP90.^58^ Our experiments show that treatment with XL-888 reduces SOX2 levels across genetically distinct GBM stem cell lines. This targeting of a core component of GBM stem cell identity may partly explain the ability of XL-888 to overcome heterogeneous GBM stem cell models.

HSP90 has been proposed to be a target for counteracting multiple oncology drug resistance mechanisms.^59^ However, several drug resistance mechanisms induced in response to HSP90 inhibition have recently been uncovered, which may limit the clinical efficacy of existing and future HSP90 inhibitors. These mechanisms include activation of the heat shock transcription factor HSF1 that is considered to limit the activity of HSP90 inhibitors through HSF1-dependent transcriptional induction of HSP70, HSP27, and to some degree HSP90 itself.^60^ Silencing of either HSP70 or HSP27 has been shown to dramatically increase cancer cell sensitivity to HSP90 inhibition and induction of apoptosis, thus combining HSP90 inhibitors with inhibitors of HSF1, HSP70, and HSP27 may enhance efficacy.^61^ The upregulation of alternative HSP90 isoforms such as GRP94 (endoplasmic reticulum) or TRAP1 (mitochondria) can sustain chaperone activity for oncoproteins despite inhibition of cytosolic HSP90α/β, thus drug combination or polypharmacology strategies targeting multiple HSP90 isoform may show enhanced long-term efficacy over isoform specific inhibitors.^62^ HSP90 inhibitor resistance and upregulation of the transcription factor Nuclear Factor Erythroid 2-Related Factor 2 (NRF2) are closely linked, with NRF2 promoting resistance by activating antioxidant and cell survival genes. Conversely, specific HSP90 inhibitors can be metabolized into more potent compounds by NRF2-activated enzymes, creating a synthetic lethal interaction where dual NRF2 and HSP90 inhibition is more effective.^63^ HSP90 activity has previously been shown to act as a buffer for proteotoxic stress such as that induced by proteasome inhibition. Thus, exploitation of proteotoxic stress dependence in combination with proteasome inhibitors may enhance efficacy.^64^

To the best of our knowledge, XL-888 has not been previously identified as a candidate against GBM. XL-888 has three hydrogen-bond donors and exhibits a relatively high molecular weight (503.6 Da) and *c* log *P* (3.56), which are suboptimal features to cross the blood–brain barrier. However, its modular synthesis^42^ together with its high potency against GBM cells makes XL-888 a promising candidate for chemical fine-tuning into a brain-penetrant anti-GBM agent. In addition, XL-888 has been shown to have protective effects on brain endothelial cells,^65^ which suggests a promising safety profile. Our results therefore suggest XL-888 as a candidate for further medicinal chemistry campaigns. We propose some structural changes to improve its physicochemical properties while maintaining its bioactivity (SI Fig. 5 and Table 1).

In summary, here we described a combined machine learning and high throughput screening approach that provides substantial efficiency gains in identifying compounds that overcome glioblastoma stem cell heterogeneity. Applications of this approach to other diverse chemical libraries may yield more novel chemical starting points and their mechanisms which have the ability to target glioblastoma stem cell heterogeneity. The approaches described here could also be applied together with other disease relevant phenotypic screening assays to identify clinical candidates, chemical starting points and target-mechanisms that address multiple complex disease areas of unmet medical need.

## Methods

### Cell culture

Patient-derived GBM stem cell lines GCGR-E13, GCGR-E21, GCGR-E28, GCGR-E31, GCGR-E34, and GCGR-E57 were obtained from the Cancer Research UK Glioma Cellular Genetics Resource, Edinburgh. For details of the cell culture protocol for these GBM stem cell lines, please refer to Materials and Methods section from Elliott *et al.*^24^

### Western blot

Cells were washed with ice-cold PBS and lysed in RIPA buffer (50 mM Tris–HCl pH 8, 150 mM NaCl, 1% Triton X-100, 0.5% sodium deoxycholate, 0.1% SDS) supplemented with protease and phosphatase inhibitor cocktails (Roche). Lysates were incubated for 20 min at 4 °C, clarified by centrifugation at 14 000×*g* for 15 min, and protein concentrations determined using the Bradford assay reagent (Thermo Fisher). Equal amounts of protein (20–30 µg) were mixed with Laemmli buffer, denatured at 95 °C for 5 min, resolved on 4–15% SDS-PAGE gels (Bio-Rad), and transferred to nitrocellulose membranes. Membranes were blocked in 5% Non fat dry milk/TBS-T (0.1% Tween-20) for 1 h at room temperature, incubated with primary antibodies overnight at 4 °C, washed, and then probed with HRP-conjugated secondary antibodies for 1 hour at room temperature. Signals were detected using enhanced chemiluminescence (Clarity ECL, Bio-Rad) and imaged on a ChemiDoc MP system. GAPDH (cell signaling, 2118) or α-Tubulin (cell signaling, 3873) served as loading controls.

### Compound screening

Cells were seeded onto laminin coated, 384 well plates (Greiner Bio-One, uClear, 781 091) and incubated for ∼20 h prior to compound treatments for 72 h. Five libraries were used for screening, giving in total 3856 compounds. Their concentrations and number of molecules per library are shown in Fig. 1A. Out of the five channels of the original cell painting assay, only the stain for the nuclei was used in this manuscript (stain Hoechst 33 342). For further details regarding this screen, please refer to Elliott *et al.*^24^

### Compounds for validation

The specifics of the compounds used in this study are: AT13387 (STRATECH SCIENTIFIC Ltd, A4056-APE-5mg), BCC0030597 (Mcule, P-35171523), conglobatin (STRATECH SCIENTIFIC Ltd, C7852-52-USB-500µg), CP-673451 (B2173-APE-5mg), PF-04929113 (STRATECH SCIENTIFIC Ltd, A4065-APE-5mg), SN-38 (Molport, 002-317-317), XL-888 (STRATECH SCIENTIFIC Ltd, A4388-APE-5mg).

### Image acquisition and analysis

Plates (384w, Greiner Bio-One, #781091) were imaged using an ImageXpress-Confocal Ht. ai high content microscope (Molecular Devices). The images were custom-analysed with the software CellProfiler v3.1.5 (https://cellprofiler.org/). For full details on the image acquisition and analysis, please refer to Elliott *et al.*^24^ Dose response validation and cell cycle effects of the selected compounds was carried out in triplicate (minimum *n* = 3 biological replicates) across 6 GSC cell lines *via* quantification of stained nuclei (Hoechst-33342) using in-built ‘Count Nuclei’ module (MetaXpress Software version 6.7.2.290, Molecular devices). Wells were imaged with 20× objective across 16 sites (∼52% well coverage). Cell cycle quantification was carried out from DNA content analysis (Hoechst stain) *via* MetaXpress ‘Cell Cycle’ module (custom gating for each cell line, using DMSO images for training). Exported data was further plotted using GraphPad Prism (v10.2.0).

### Hit validation

We queried the 100 XGboost models with all compounds in the screening library to produce predicted probabilities of compound efficacy. Predicted hits from the computational screen were selected using a cutoff of 22% average probability across all 100 models. This led to 7/12 888 compounds in the screening library. For hit validation, we employed the same six cell lines that were used for generating the training data (see above). Dose–response curves are shown in Fig. 3C–E for the three compounds that displayed activity against GBM cell lines. Mean ± s.d. are shown from *n* = 3 experiments. All experimental data is available in the data source file.

### Live cellular imaging

Compound induction of apoptosis over time was quantified using an Incucyte SX5 (Sartorius) live cell imaging microscope. GCGR-E21 and -E57 cells were seeded (1000 and 500 cells/well, respectively) on laminin coated 384-well plates (10 µg mL^−1^). After ∼24 h of incubation, NucView-488 caspase-3 detection reagent (5 µM, Biotium) was added. The cells were subsequently dosed as indicated, using a D300 digital dispenser (Tecan), and loaded onto an IncuCyte SX5 live imaging platform. Staurosporine (200 nM) was used as a positive control for apoptotic induction *vs.* DMSO (0.1% v/v) as negative control. Images were taken at 3 h intervals for ∼120 h. Fluorescent apoptotic cell bodies were quantified as FU ‘Counts per image’ using IncuCyte software analysis modules. Exported data was further plotted using GraphPad Prism (v10.2.0).

### Network analysis

For the three experimentally-validated hits, we obtained a predicted target list with a similarity ensemble approach (SEA^39^). This software queries the SMILES string of an added compound to query potential compound–protein interactions across its database, taking into consideration species, Tanimoto coefficient (MaxTC) and *p*-value. The predicted targets were then fed into the STRING platform to assess the representation of the network components in biological pathways (KEGG database^66^) and biological processes (Gene Ontology^67^) that may be influenced by each compound.

### Data assembly for model training

To make optimal use of the compound screening data across different concentrations and heterogeneous GBM stem cell lines, we labelled compounds as positive or negative with a concentration- and cell line-dependent approach. For compounds that impacted nuclei count at only one concentration, we chose said concentration. For those that impacted nuclei count at more than one concentration, we chose the concentration in which the effect was closest to 50% nuclei count. Compounds that did not affect nuclei counts at any concentration were labelled as negative. After choosing a single concentration per compound, they were labelled as positive if they caused a decrease in nuclei count of 35% with respect to the DMSO controls in at least two, but not all, cell lines. The rest of compounds were labelled as negative. The resulting training data contained 3458 positives and 103 negatives.

### Physicochemical visualisations, model training and computational screen

For model training and virtual screening, compounds were featurized with 200 physicochemical descriptors computed with the RDKit package^68^ on the SMILES strings after salt removal. The majority of SMILES were taken from the library of origin of every compound (C3L, KCGS, LOPAC, Prestwick, and TargetMol anticancer-330 for training; BioAscent-3000 and TargetMol anticancer-330 for screening). For chiral molecules, we employed isomeric SMILES. Tanimoto distances were calculated using custom R code.

Machine learning models were trained with the python libraries scikit-learn 1.3.0 and XGBoost 1.7.3. We first removed 11 features from the 200 physicochemical descriptors because they had a constant value for all compounds in the training set. We then reduced the number of features using scikit-learn feature importance with a forest of trees function and the average reduction of Gini index as an impurity measure.^69^ Models were then trained on a reduced set of 172 *z*-score normalised features with non-negative importance scores. We retrained and tested XGBoost models on resampled 70 : 30 data splits (stratified with *N* = 2493 for training and *N* = 1068 for testing), until we obtained 100 models with test accuracy above the baseline given by the class imbalance (1 − 103/3561 = 0.971). Model hyperparameters were manually calibrated *via* grid search using accuracy as performance metric (learning_rate = 0.5, max_depth = 2, *n*_estimators = 100, and colsample_bytree = 0.5), with all other hyperparameters set to default values. Each of the 100 models that surpassed the naive baseline was trained with the same hyperparameters listed above and each was independently used to virtually screen the BioAscent-3000 and TargetMol anticancer-330 libraries (Fig. 3B). Models were scored with precision (TP/(TP + FP)), recall (TP/(TP + FN)), *F*1-score (geometric mean of precision and recall), accuracy, and the areas under the Receiver Operating Characteristic and Precision–Recall curves.

## Author contributions

V. S. B. designed computational modelling approach, curated datasets, created data visualisations, performed and evaluated virtual screen; R. J. R. E. performed compound screening, lead compounds characterisation and data analysis, performed *in vitro* research and experiments of mechanism of action; J. C. D. performed compound screening and data analyses; M. F. performed experiments of mechanism of action and stemness, A. L. M. and A. U. B. contributed with structural compound analyses; D. A. O. and N. O. C. designed and supervised the research. All authors wrote the original draft and contributed to subsequent editions.

## Conflicts of interest

The authors declare no conflict of interests.

## Supplementary Material

DD-005-D5DD00190K-s001

## Data Availability

Training and screening data have been deposited in Zenodo at https://doi.org/10.5281/zenodo.17100377. Code availability: python code for model training and compound screening has been deposited in Zenodo at https://doi.org/10.5281/zenodo.17100377. Supplementary information (SI): representative images of the six glioma stem cell lines used in this work; the experimetal results concern HSP90 inhibitors' studies of apoptosis induction, cell cycle and stemness effects; a structural and physicochemical properties analysis of the compound XL-888. See DOI: https://doi.org/10.1039/d5dd00190k.
