# Supplementary material for: Identification of drug candidates against glioblastoma with machine learning and high-throughput screening of heterogeneous cellular models
Source: Digit Discov. 2026 May 13;5(6):2560–74. doi: 10.1039/d5dd00190k (PMC13181845; doi:10.1039/d5dd00190k)
Supplement: DD-005-D5DD00190K-s001 [file DD-005-D5DD00190K-s001.pdf]

Supplementary Information (SI) for Digital Discovery.
This journal is © The Royal Society of Chemistry 2026

Supplementary Material

Identification of drug candidates against glioblastoma with machine learning and high-throughput screening of heterogeneous cellular models

Vanessa Smer-Barreto[1,2], Richard J. R. Elliott[1], John C. Dawson[1], Álvaro Lorente-Macías[1], Muhammad Furqan[1], Asier Unciti-Broceta[1], Diego A. Oyarzún[2,3,*], Neil O. Carragher[1,*]

[1]Cancer Research UK Scotland Centre, Institute of Genetics and Cancer, University of Edinburgh, Crewe Road South, Edinburgh, EH4 2XR, UK.
[2] School of Informatics, University of Edinburgh, 10 Crichton St, Edinburgh, EH8 9AB, UK.
[3] School of Biological Sciences, University of Edinburgh, Max Born Crescent, Edinburgh, EH9 3BF, UK.
* Corresponding authors: d.oyarzun@ed.ac.uk; n.carragher@ed.ac.uk

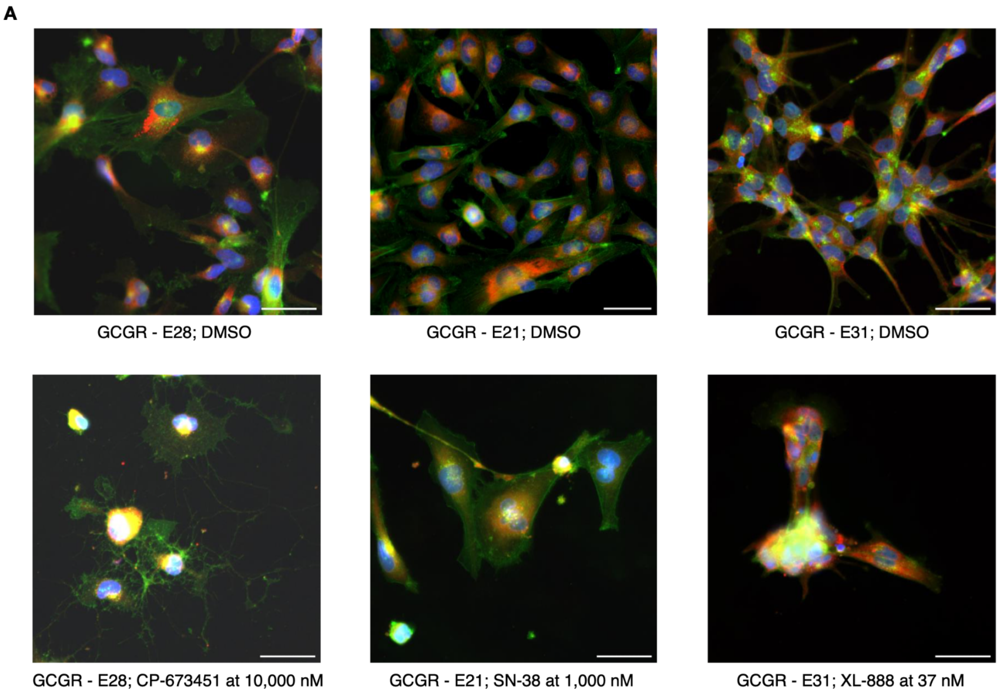

Supplementary Figure 1. Representative images of cell line responses to compound administration vs DMSO controls. (**A**) Top row: DMSO controls. Bottom row: CP-673451 at 10,000 nM for cell line GCGR-E28 of classical GBM subtype, SN-38 at 1,000 nM for cell line GCGR-E21 of mesenchymal GBM subtype, and XL-888 at 37 nM for cell line GCGR-E31 of proneural GBM subtype.

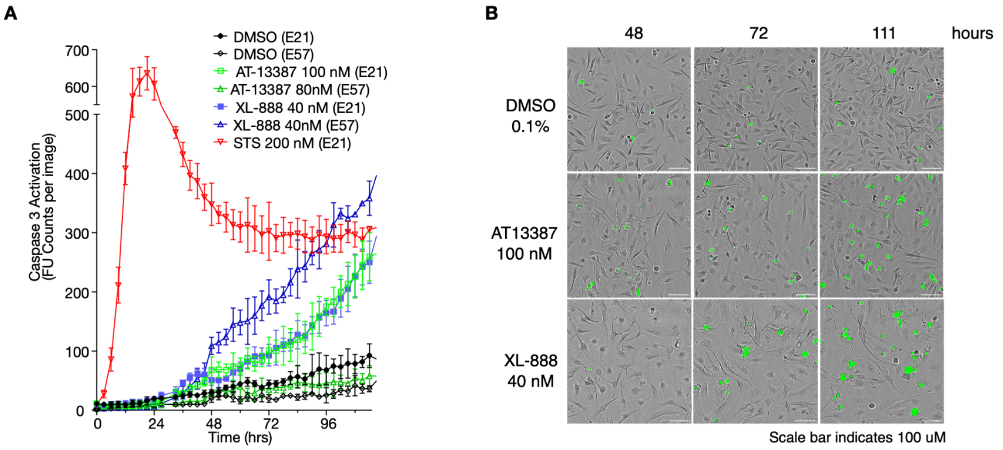

Supplementary Figure 2. Incucyte Live Cell Imaging; Induction of apoptosis by XL-888 and AT-13387 over time. (**A**) Plot of Caspase 3 activation over time (hours) quantified as FU Counts per image (apoptotic cells) on GCGR-E21 & -E57 cells (mesenchymal subtypes). Staurosporine (200nM, Red) was used as positive control for apoptosis (STS on E21 cells shown only). DMSO (0.1% v/v) was used as a negative/vehicle control for cell death. AT-13387 (green) & XL-888 (blue) were assayed at varied concentrations (close to IC_{50} values). (**B**) Representative live cell images of GCGR-E21 cells showing progression of apoptosis (green cell mask) over 48, 72 and ~111 hours, induced by XL-888 (40 nM) and AT13387 (100 nM), compared to DMSO controls.

A

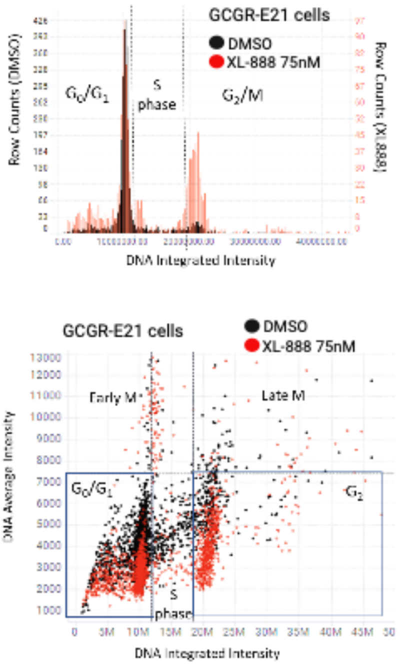

B

	DMSO	XL888 75nM
G_0/G_1 %	76.3	52.2
S-phase %	15.7	20.2
G_2/M %	8.0	27.6
total cells	*3250*	*1528*
G_0/G_1	*2481*	*798*
S-phase	*509*	*308*
G_2/M	*260*	*422*

C

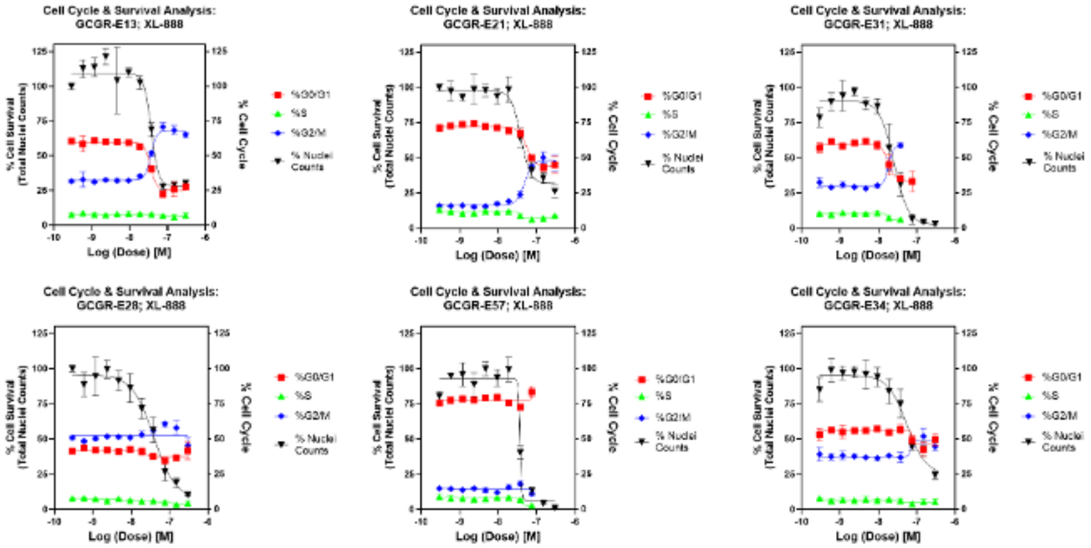

D

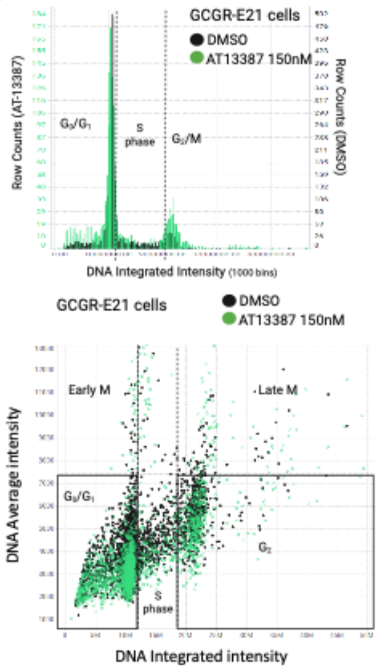

E

	DMSO_2	AT-13387 150nM
**G_0/G_1 %**	75.5	65.9
**S-phase %**	14.2	14.3
**G_2/M %**	10.3	19.8
total cells	*4283*	*2295*
G_0/G_1	*3233*	*1512*
S-phase	*608*	*328*
G_2/M	*442*	*455*

F

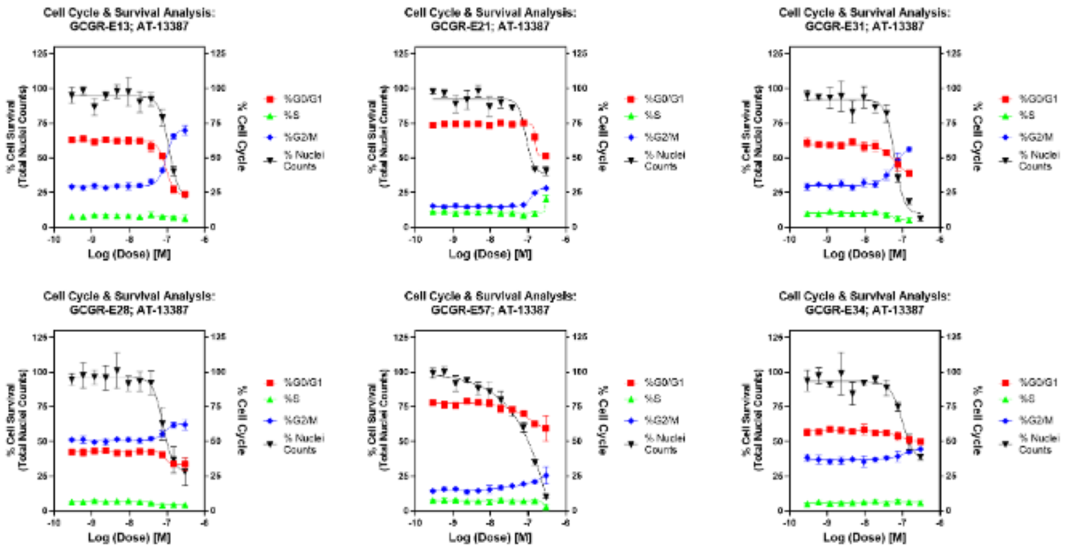

Supplementary Figure 3. Cell Cycle effects of HSP90 inhibitors on glioma stem cells from image based-DNA content analysis. (**A**) Representative histogram (DNA Integrated Intensity) and scatter plot (DNA Average Intensity vs Integrated Intensity) from single cell DNA content image analysis, comparing DMSO (black) wells to XL-888 (red). Cell Cycle phases are indicated (n=3, 16 sites per well, 20X objective). (**B**) Example of (single cell) data of cell cycle effects on E21 cells, using XL-888 AT 75 nM vs DMSO. Percentage (%) cell cycle populations and total cells are indicated (n=3 combined single cell data). (**C**) Percentage (%) cell cycle and percentage (%) cell survival by XL-888 dose response across 6 glioma stem cell lines. Data represents image-level, well-aggregated (average, n=16 sites) across replicates (n=3). (**D**) Representative histogram (DNA Integrated Intensity) and scatter plot (DNA Average Intensity vs Integrated Intensity) from single cell DNA content image analysis, comparing DMSO (black) wells to AT-13387 (red). Cell Cycle phases are indicated (n=3, 16 sites per well, 20X objective). (**E**) Example of (single cell) data of cell cycle effects on E21 cells, using AT-13387 150 nM vs DMSO. Percentage cell cycle populations and total cells are indicated (n=3; combined single cell data across replicates). (**F**) Percentage (%) cell cycle and percentage (%) cell survival by AT-13387 dose response across 6 glioma stem cell lines. Data represents Image level, well aggregated (average, n=16 sites) across replicates (n=3).

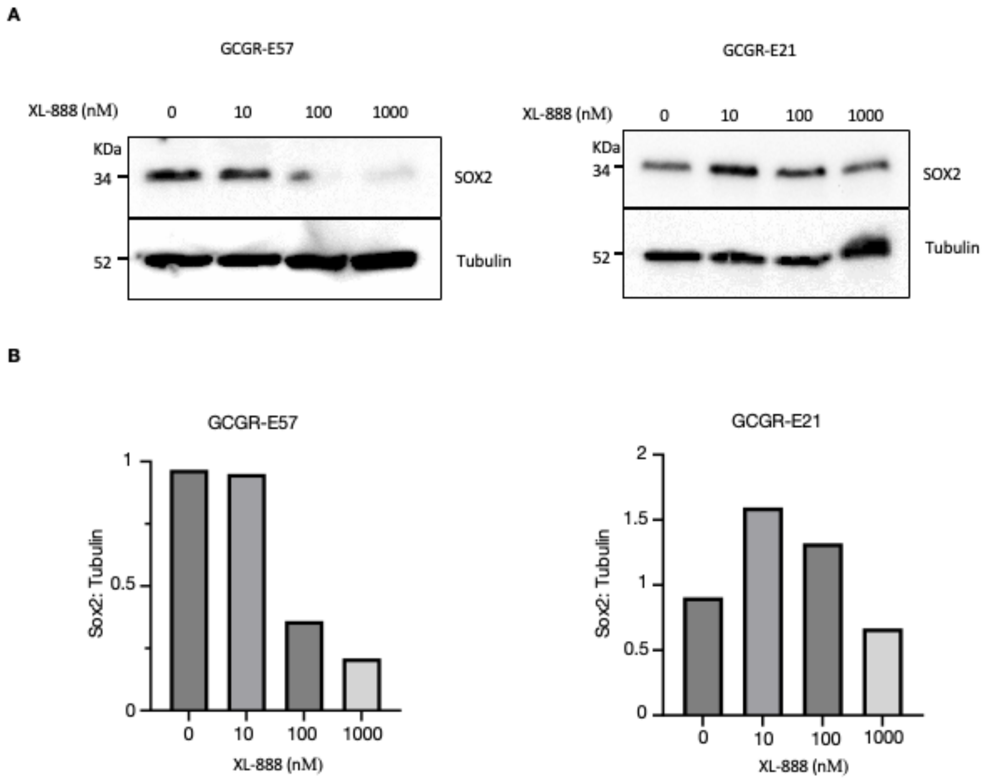

Supplementary Figure 4. Stemness effects of XL-888. (**A**) Western blot analysis of stemness marker SOX2 with Tubulin as a loading control across two GBM stem cell lines (GCGR-E57 and GCGR-E21). (**B**) Quantification of Sox2: Tubulin ratio.

A

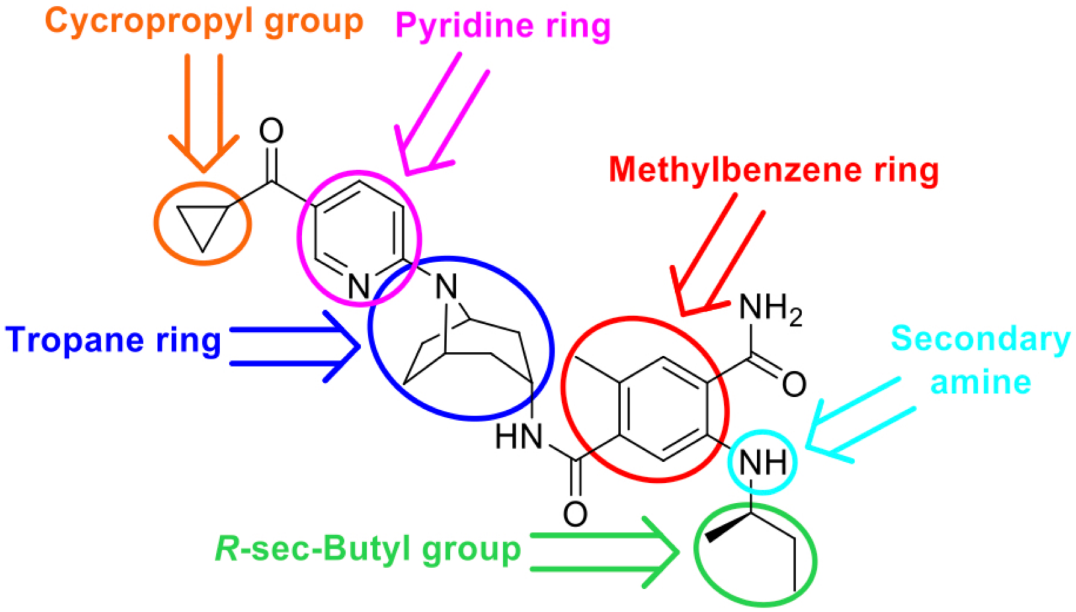

Supplementary Figure 5. Structure of XL-888. (**A**) Proposed areas to modify are highlighted with coloured circles. Reduction of the size and lipophilicity of the molecule by modifying the central tropane ring, as well as the cyclopropyl and *R*-sec-butyl moieties, which are significant contributors to the overall lipophilicity of XL-888. Exploration of the pyridine and methylphenyl rings by incorporating polar groups or by replacing them with bioisosteric heterocycles could also enhance blood-brain barrier penetration. Furthermore, the secondary amino group is another group that could be changed to improve the physicochemical properties of XL-888. While this amino has been reported to enhance cellular activity, it does not interact with any residue of HSP90[42]. Instead, it forms an intramolecular hydrogen bond with the carbonyl group of the benzamide ring. This interaction presents an opportunity for further medicinal chemistry optimization, potentially leading to derivatives with significantly improved blood-brain barrier permeability.

Table 1. XL888 physicochemical, PK data and predictions

Molecular weight	503.65 $g \cdot mol^{-1}$
Num. rotable bonds	9
Num. H-bond acceptors	6
Num. H-bond donors	3
TPSA[a]	117.42 ! ^{2}
cLogP[a]	3.56
Predicted solubility (pH = 7.4) [b]	>10 "g/mL
Papp (A-to-B, MDCK) [c]	320 # nm/s
BBB penetrant[a]	No
Intestinal absorption (rat, % absorbed)[c]	30%
CL_{int} (rat)[c]	920 mL/h/Kg
$t_{1/2}$ (rat, IV)[c]	1.4 h

[a] SwissADME. [b] DruMAP. [c] Bioorg. Med. Chem. Lett. 22 (2012) 5396–5404

Supplementary Table 1. Physicochemical properties and PK predictions of compound XL-888.
